# Rapid, field-deployable method for collecting and preserving plant metabolome for biochemical and functional characterization

**DOI:** 10.1371/journal.pone.0203569

**Published:** 2018-09-06

**Authors:** Sarah A. Skubel, Vyacheslav Dushenkov, Brittany L. Graf, Qingwei Niu, Alexander Poulev, Hetalben M. Kalariya, Llewellyn C. Foxcroft, Ilya Raskin

**Affiliations:** 1 Department of Plant Biology, Rutgers, The State University of New Jersey, New Brunswick, New Jersey, United States of America; 2 Hostos Community College, City University of New York, Bronx, New York, United States of America; 3 Centre for Invasion Biology (C•I•B), Department of Botany and Zoology, Stellenbosch University and Scientific Services, South African National Parks, Skukuza, South Africa; Universidade do Porto, Faculdade de Farmácia, PORTUGAL

## Abstract

Study of plant metabolome is a growing field of science that catalogs vast biochemical and functional diversity of phytochemicals. However, collecting and storing samples of plant metabolome, sharing these samples across the scientific community and making them compatible with bioactivity assays presents significant challenges to the advancement of metabolome research. We have developed a RApid Metabolome Extraction and Storage (RAMES) technology that allows efficient, highly compact, field-deployable collection and storage of libraries of plant metabolome. RAMES technology combines rapid extraction with immobilization of extracts on glass microfiber filter discs. Two grams of plant tissue extracted in ethanol, using a specially adapted Dremel^®^ rotary tool, produces 25–35 replicas of 10 mm glass fiber discs impregnated with phytochemicals. These discs can be either eluted with solvents (such as 70% ethanol) to study the metabolomic profiles or used directly in a variety of functional assays. We have developed simple, non-sterile, anti-fungal, anti-bacterial, and anti-oxidant assays formatted for 24-multiwell plates directly compatible with RAMES discs placed inside the wells. Using these methods we confirmed activity in 30 out of 32 randomly selected anti-microbial medicinal plants and spices. Seven species scored the highest activity (total kill) in the anti-bacterial (bacteria from human saliva) and two anti-fungal screens (*Fusarium* spp. and *Saccharomyces cerevisiae*), providing functional validation of RAMES technology. RAMES libraries showed limited degradation of compounds after 12 months of storage at -20°C, while others remained stable. Fifty-eight percent of structures characterized in the extracts loaded onto RAMES discs could be eluted from the discs without significant losses. Miniaturized RAMES technology, as described and validated in this manuscript offers a labor, cost, and time-effective alternative to conventional collection of phytochemicals. RAMES technology enables creation of comprehensive metabolomic libraries from various ecosystems and geographical regions in a format compatible with further biochemical and functional studies.

## Introduction

Natural products, particularly those derived from plants, have made invaluable contributions to human civilization. They enabled the development of human medicines, crop protection chemicals, dietary supplements, cosmetics, preservatives, disinfectants, flavors, fragrances, and colorants [1,2]. Up to 70% of all drugs in the market today have an origin or inspiration from nature [3]. The first commercially produced pharmaceutical, aspirin, synthesized by Bayer in 1897, was derived from the plant natural product salicylic acid, and, to this date, the majority of painkillers and chemotherapeutic agents originate from plants [2]. There are at least 100,000–200,000 small-molecule compounds produced by plants that are known [2–4], and this number is increasing annually.

Substantial advances in analytical instrumentation has accelerated the rate of natural product discovery in recent years; however, natural product-based formulations, particularly medicines, are facing increasing competition from chemically synthesized compounds and biologics [3, 5]. Plant-based natural product discovery and research are also hindered by worldwide destruction of natural habitats, disappearance of many species, as well as political and logistical concerns associated with the 1992 Rio Convention on Biological Diversity [6, 7].

Field collection and functional testing of plant metabolites generally involves harvesting plant material, drying it in the sun or hot air, extracting the metabolites using solvents in a single or multi-step process, and then drying the extract in containers from which subsamples can be taken for future use. Libraries produced in this way require destructive and laborious harvesting, large volumes of solvents, significant amounts of preparation time, and extensive storage space. The time required to collect, grind, extract and dry plant materials produced this way facilitates degradation of unstable phytochemicals and formation of unnatural metabolites [8]. These traditional collection methods make the preparation of plant metabolomic libraries laborious, expensive and logistically complicated, especially in comparison to synthetic and combinational libraries. These factors contribute to a downward trend in developing drugs from plants and other natural sources, despite their lasting potential as a source of bioactives for future pharmaceuticals [9–11]. In contrast, the rest of the consumer product industry is experiencing an ever-growing demand for natural, organic and green products and technologies [12,13].

Cataloging and preserving plant metabolome is critically important at a time when Earth is facing the catastrophic loss of biodiversity, equaled to a sixth mass extinction [14]. Recent estimates suggest that there are 450,000 vascular plant species (flowering plants, conifers, ferns, mosses and liverworts) on the planet, two thirds of which are found in the tropics. One third of all plant species are at risk of extinction, disappearing at 1,000 to 10,000 times the background rate [15]. The extinction of species and their biochemical diversity represents an irreplaceable loss for our planet.

There is a need for more efficient technologies providing a sustainable, compact and cost-effective format for preserving and cataloging plant biochemical diversity that are compliant with the Rio Convention. Metabolomic libraries of the future should be produced in a format that allows long-term storage in the countries of origin, with minimum need for space, curation, and maintenance. Moreover, these libraries should be coupled with a vouchering system, allowing reliable taxonomic attribution of all samples.

The methodology presented in this manuscript describes a rapid and cost-effective method of plant metabolome collection and preservation, which is compatible with functional screening and research. This method enables collecting and storing plant metabolomic libraries in the countries of origin with limited workforce, training, and resources. It is a fully field-deployable method requiring only two grams of plant material. We also present data that support the stability of the collected libraries and demonstrates their use and compatibility with functional assays.

## Materials and methods

### Ultra-performance liquid chromatography—mass spectrometry metabolome analysis

The UPLC/MS metabolome analysis system consisted of the Dionex^®^ UltiMate 3000 RSLC ultra-high pressure liquid chromatograph, workstation equipped with the ThermoFisher Scientific’s Xcalibur v. 4.0 software package combined with Dionex^®^’s SII LC control software, solvent rack/degasser SRD-3400, pulseless chromatography pump HPG-3400RS, autosampler WPS-3000RS, column compartment TCC-3000RS, and photodiode array detector DAD-3000RS. After the photodiode array detector the eluent flow was guided to a Q Exactive Plus Orbitrap high-resolution high-mass-accuracy mass spectrometer (MS) (Thermo Scientific^TM^, Waltham, MA). Mass detection was a full MS scan from 100 to 1000 m/z in either positive, or negative ionization mode with electrospray (ESI) interface. Sheath gas flow rate was 30 arbitrary units, auxiliary gas flow rate was 7, and sweep gas flow rate was 1. The spray voltage was 3500 volts (-3500 for negative ESI) with a capillary temperature of 275°C. The mass resolution was 140,000 m/Δm FWHM. Compounds were separated on a Phenomenex^TM^ Kinetex C8 reverse phase column, size 100 x 2 mm, particle size 2.6 mm, pore size 100 Å. The mobile phase consisted of 2 components: Solvent A (0.5% ACS grade acetic acid in LCMS grade water, pH 3–3.5), and Solvent B (100% Acetonitrile, LCMS grade). The mobile phase flow was 0.20 ml/min, and a gradient mode was used for all analyses. The initial conditions of the gradient were 95% A and 5% B; for 30 min the proportion reaches 5% A and 95% B, which was kept for the next 8 minutes, and during the following 4 min the ratio was brought to initial conditions. An 8 min equilibration interval was included between subsequent injections. The average pump pressure using these parameters was typically around 3900 psi for the initial conditions.

Putative formulas were determined by performing isotope abundance analysis on the high-resolution mass spectral data with Xcalibur v. 4.0 (Thermo Scientific^TM^, Waltham, MA) software and reporting the best fitting empirical formula. Database searches were performed using the Reaxys.com (RELX Intellectual Properties SA) and SciFinder (American Chemical Society). The databases were reviewed for compounds identified from the analyzed genera with molecular masses corresponding to the LC-FTMS data. Any matches were investigated by comparing the literature and the experimental data; putative compound assignments were made when matches were identified.

### Anti-fungal assays

Anti-fungal assays were carried out in CELLSTAR “R” Cell Culture Multiwell Polystyrene Greiner Bio-One 24-well plates. *Fusarium* spp. were isolated from locally purchased Idaho potatoes. Excised potato disc, with skin, was placed in a Petri dish containing media prepared from 24 g/L potato dextrose, 15 g/L agar and 150 mg/L spectinomycin. Taxonomic identification of *Fusarium* spp. was performed under a light microscope and confirmed by Dr. James White, Rutgers University. Once isolated *Fusarium* hyphal growth covered the inoculated Petri dish, it was ready for use in the assays. For *Fusarium* assays, each well on a plate was filled with 600 μl of LB (Miller) broth (Sigma Life Science) with spectinomycin (0.15 g/L) to curtail bacterial growth. Dried baker’s yeast (*Saccharomyces cerevisiae*) was purchased locally. Yeast inoculum solution was prepared by adding 250 mg of yeast powder and 250 mg of table sugar to 5 ml of Millipore water, vigorously shaking the mixture and then allowing it to settle for 5–10 min.

### Anti-bacterial assays

Anti-bacterial assays were performed in Multiwell Polystyrene Greiner Bio-One 24-well plates. We have observed that human saliva serves as an excellent inoculum of easily culturable bacteria for Screens-to-Nature (STN) screens. This inoculum is always available in the field and contains a wide array of human-associated bacteria, including some opportunistic pathogens [16,17]. To prepare saliva inoculum, human saliva was collected in a test tube or vial and 50 *μ*l of fresh saliva was added to each well containing 600 μl of LB (Miller) broth. Saliva inoculum can be diluted with 1–3 volumes of water.

### Viability staining with MTT

The effect of phytochemicals on both fungal and bacterial growth can be visually assessed as the appearance of white *Fusarium* hyphi in the wells or development of turbidity (cloudiness) associated with yeast and bacterial growth. However, staining with MTT [3-(4, 5-dimethylthiazole-2-yl)-2, 5- diphenyltetrazolium bromide] at 5 mg/ml is a more reliable, quantitative and qualitative, color indicator of anti-fungal and anti-bacterial activities. Live fungi and bacteria utilize dehydrogenases to convert yellow-colored MTT to a dark purple formazan [18]. Thus, the presence of anti-fungal or anti-bacterial activity could be detected 2 h following MTT addition by the color of the solution inside the wells. Light yellow color indicates dead cells and high activity, while a change to dark purple color indicates living cells and little activity.

### Anti-oxidant assays

Anti-oxidant activity was determined with standard ABTS [2, 2 azino-bis (3-ethylbenzo-thiazoline-6-sulfonic acid)] [19] assay in a 24-multiwell STN format. Each well was filled with 600 μl of freshly prepared assay solution containing 7 mg/ml ABTS in water, to which 20 μl of 0.5 mg/ml potassium persulfate (K_2_S_2_O_8_) solution from freshly prepared 50 mg/ml stock is added.

## Results

### Collection of plant metabolome samples

The RApid Metabolome Extraction and Storage (RAMES) method for collecting and storing metabolomic libraries of phytochemicals is designed for speed, efficiency, low-cost, simplicity, portability, long-term storage, and compatibility with bioactivity assays. It also allows sustainable sample collection that does not destroy a source plant. Every step can be operated in the field, without a need for a wet laboratory or constant power supply. Simple power tools used for collection use rechargeable batteries that can be recharged from the USB or cigarette lighter port of any vehicle.

**Fig 1:**
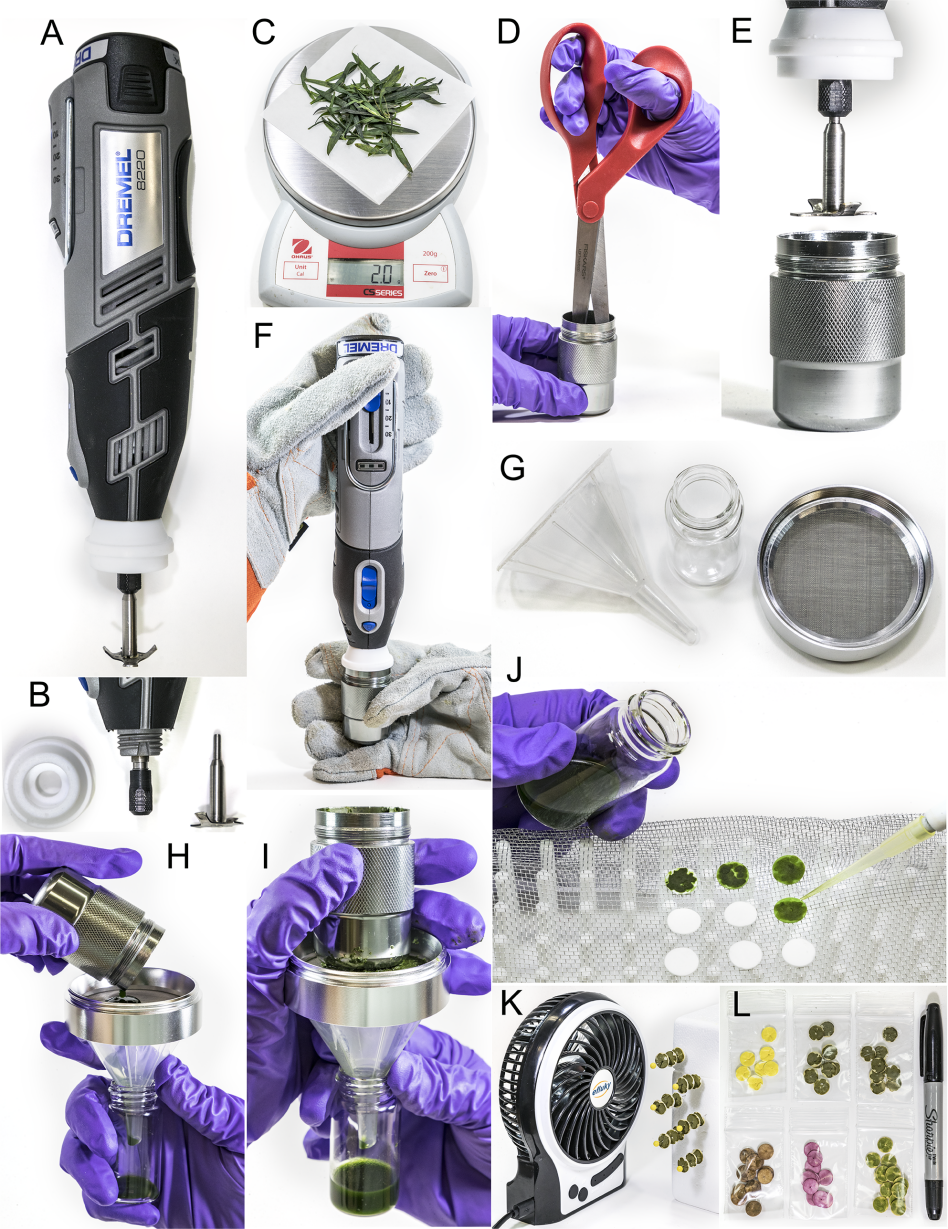
Illustration of RAMES method of preparing metabolome libraries. (See text for details).

**Fig 2:**
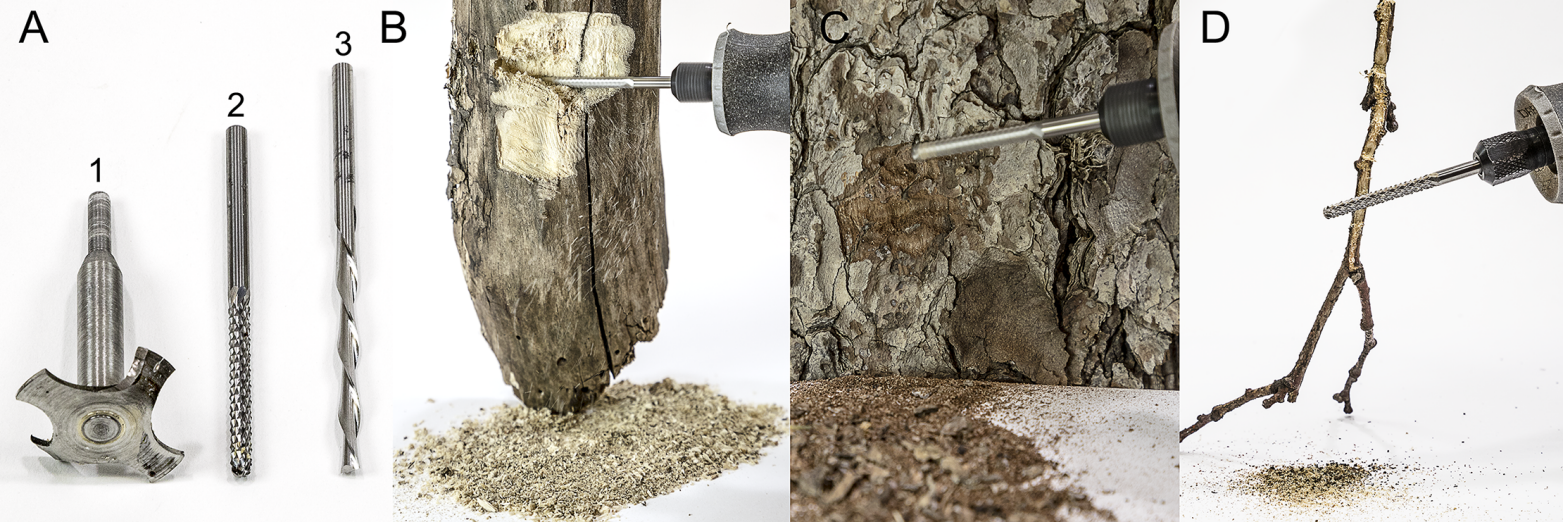
Illustration of RAMES method of grinding hard plant tissues prior to extraction. A1, Specially manufactured bit for grinding and extracting plant tissues (also shown in Fig 1A, 1B and 1E); A2, Dremel 562 tile grinding bit; A3, Dremel 561 multipurpose bit. B, Rapid grinding of woody stems to produce small particles suitable for extraction; C, Grinding of bark; D, Grinding of branches.

Recording GPS coordinates of the collection site and taking a photo of the plant before collection is strongly recommended, as well as noting the time of collection, weather, topography, physiological and developmental characteristics of the collected tissue, and other characteristics of the collection site and sampled plant. Grinding and simultaneous extraction of the plant tissue is performed with the specially adapted cordless, variable speed, Dremel rotary tool Model 8220, 12V or similar (Dremel, a division of Robert Bosch GmbH Co., Racine, WI) fitted with a nose cup modified to serve as a lid for the grinding chamber. A specially designed and manufactured grinding bit, with four slightly angled rectangular blades, was experimentally determined to be the most effective configuration for rapid plant extraction (Fig 1A, 1B and 1E and Fig 2A1). Always wear protective goggles and thick, safety gloves when operating the Dremel tool. Using scissors or a knife, excise two grams of tissue from the sample plant, and then further cut it into smaller pieces. Adjust the weight to 2 g on a portable, battery-operated balance (CS Series, Ohaus, Parsippany, NJ) (Fig 1C). Transfer the sample into a 35 mm diameter x 55 mm depth aluminum chamber (Hy-Ko Products Co., Super Survivor Capsule, # KB348-BKT) and shred the tissue inside the chamber with scissors or alike (Fig 1D), to 4–8 mm pieces. Add 5 ml of 95% ethanol to the chamber. This volume works well for most leaves and flowers. For particularly succulent samples with high water content (i.e. fruits or water storage organs), 4 ml ethanol is sufficient.

With one gloved hand holding the chamber and the other gloved hand securing the Dremel tool, lower the blade into extraction chamber with the nose cup lid fitting tightly over the chamber opening and blade almost reaching the bottom (Fig 1E and 1F). Turn on the Dremel tool and slowly increase the speed to near the maximum power. Slightly tilt and shake the setup while grinding to encourage tissue contact with the blade.

Fifteen to thirty seconds at full grinding speed is normally sufficient to transform the tissue and solvent into a slurry. This time may vary depending on the nature of the sample and the particle size of the starting plant material. Turn off the Dremel tool before removing the grinding blade from the chamber. Filter the slurry through a fine stainless steel mesh filter (removed from 2.5-inch Ultimate 4-piece Aluminum Golden Gate^®^ Herb Grinder) position on top of a 5 cm-wide plastic funnel inserted into a 20 ml glass scintillation vial (Fig 1G). Hold the filtration assembly vertically and slowly pour the extraction slurry on top of the stainless-steel filter (Fig 1H). Press the bottom of the extraction chamber over the slurry on top of the filter to squeeze out as much extract as possible (Fig 1I). A scintillation vial with the collected extract can be capped, labeled, and stored before final processing. However, to prevent degradation of unstable compounds, it is recommended that the extract is processed immediately.

Metabolite collection and immobilization is performed using rapid loading and drying (sorption) of the extract onto the filter discs. We have evaluated six types of commercially available filter discs made from cellulose and glass fiber (Table 1). Absorbency of 70% ethanol solution, drying time with portable fan, and content of impurities [determined by Ultra High-Pressure Liquid Chromatography / Mass Spectrometry (UPLC/MS) analysis of compounds eluted from the filtering discs with 70% ethanol], were used as key factors for selecting the most suitable filter discs for metabolome collection and preservation. Based on these properties, Whatman® glass microfiber filters, Grade GF/D (Whatman # 1823–010, purchased from Millipore Sigma,) were selected as the best sorption substrate for metabolome preservation. These discs contained almost no interfering compounds eluted with 70% ethanol and could sorb up to 90 μl of 70% ethanol before reaching saturation. GF/D is available in 10 mm diameter, an optimal size for metabolite collection and functional analysis using 24-multiwell plate format.

**Table 1:** Comparative analysis of different filter discs according to size (mm), absorbency (μl/mm^3^), drying speed (μl/s), and drying time (s).

Filter Type	Description	Diameter	Thickness	Absorbency	Drying speed	Drying time
Cellulose (Qualitative)	Grade 1	15	0.18	0.25	0.23	34
Cellulose (Qualitative)	Grade 3	23	0.39	0.49	0.98	81
Cellulose (Quantitative)	Grade 540	21	0.16	0.31	0.43	40
Glass Microfiber	GF8	25	0.35	1.78	0.60	507
Glass Microfiber	GF6	21	0.35	0.47	0.64	89
Glass Microfiber	GF/D	10	0.53	1.50	0.12	487

### Immobilizing metabolome samples on glass fiber discs

Pipette 90 μl of the filtered, liquid extracts onto a 10 mm GF/D glass fiber disc resting on top of an aluminum window screen mesh, placed on top of a test tube rack or similar support (Fig 1J). Window screen provides a good surface to support the discs and prevents extract spillage. Continue loading the RAMES discs until the extract is finished. Two grams of plant material, such as leaves, produce a minimum of 2.5–3.5 ml recoverable extract. This is sufficient for loading 25–35 GF/D 10 mm glass fiber discs. Use a long metal pin or a sturdy, thin wire to puncture the loaded discs in the center and string them along the pin (Fig 1K). To rapidly dry the RAMES discs, place the strung discs in front of a cordless, rechargeable fan (Efluky Mini USB 3 Speeds Rechargeable Portable Table Fan, 4.5-Inch). Styrofoam boxes (Fig 1L) or other soft materials provide an easy support for forced air-drying. Depending on the ambient temperature and humidity, drying takes 3–8 min. Finally, place the RAMES discs inside properly labeled zip-lock plastic bags (5 x 6.5 cm) (Fig 1M). In this format, RAMES libraries can be easily transported and stored at -20°C. Secondary containers, such as larger zip-lock bags or small cardboard boxes can be used to facilitate transport and storage. In humid climates, desiccants, such as silica gel, should be placed inside the secondary containers. A standard upright freezer can store tens of thousands of RAMES samples collected according to this protocol. While testing the RAMES technology in the field, we discovered that a heavy-duty spray bottle is an excellent water-saving device for washing the equipment used in preparing RAMES libraries in the field.

### Extracting dry and woody plant tissues

Some plant tissues are dry and hard (seeds, bark, branches, woody stems, and tree trunks), requiring modifications to the extraction process. Small seeds can be rapidly ground to a powder inside the extraction chamber using the equipment shown in Fig 1, equipped with a specially manufactured bit (Fig 2A1 and Fig 1A, 1B and 1E). Large seeds can be wrapped in a cloth and crushed with a hammer before being ground. Woody parts of a plant, such as stems (Fig 2B), bark (Fig 2C), and branches (Fig 2D), can be rapidly ground to fine particles with a Dremel 562 tile grinding bit (Fig 2A2), which produces finer particles, or a Dremel 561 multipurpose bit (Fig 2A3), which produces relatively coarser particles. Grind woody parts over a piece of paper to catch the falling particles, and then fold the paper into a funnel to transfer the ground material onto a balance and into a grinding chamber. Dry plant material, such as wood or bark, absorbs large volumes of solvent, thus only 200 mg of dry ground material in 5 ml of 70% ethanol is needed to produce extracts for RAMES libraries. Extract the resulting slurry for 15–30 seconds using the standard Dremel extraction process (Fig 1) and the grinding / extraction bit (Fig 2A1). It takes less than one minute to produce 200 mg of finely ground plant material suitable for extraction. As an optional step, which improves the extraction efficiency, the grinding chamber with a slurry can be capped and incubated for 10–30 min pre-filtration. Subsequent processing of the extracts is done as shown in Fig 1H–1M. Seeds or other, less hygroscopic, dry tissues may be extracted in larger sample sizes, i.e. 1–2 g, in 5 ml of 70% ethanol, to produce sufficient volumes of extract for loading RAMES discs.

### Vouchering and genotyping

DNA barcoding of variable DNA regions is becoming a popular, accepted method for plant identification, limited mainly by the availability of sequencing information for all plant species and, in some cases, lack of variability of sequence variation at a species levels [20,21]. While herbarium vouchers provide the most reliable method of confirming taxonomic identity, collecting a herbarium voucher is impractical in many field situations, as it requires significant amount of time, equipment, storage space, and plant material. Therefore, collecting a small tissue sample (less than 1 g) for DNA barcoding along with RAMES samples offers a viable strategy for confirming plant identification, should it be necessary in the future. A rapid method for field collecting plant samples for DNA studies and barcoding, using silica gel as a drying method has been developed and tested [22]. The method involves placing a small tissue sample under 1 g in weight, such as a leaf disc 10 mm in diameter, in a zip-lock bag with silica gel desiccant laced with moisture indicator. Rapid tissue desiccation effectively preserves DNA for future genetic analysis and can be performed simultaneously with collecting RAMES libraries. Barcoding samples could be stored in the same zip-lock bags with corresponding RAMES samples. We recommend collecting 2–3 barcoding samples to complement a RAMES library sample. We also recommend taking detailed, close-up photographs of harvested plants that can be used to confirm taxonomic identification in the absence of herbarium voucher. Photographs of flowers and fruits are especially important for the identification of species with similar vegetative structures.

### RAMES stability and sample recovery

**Fig 3:**
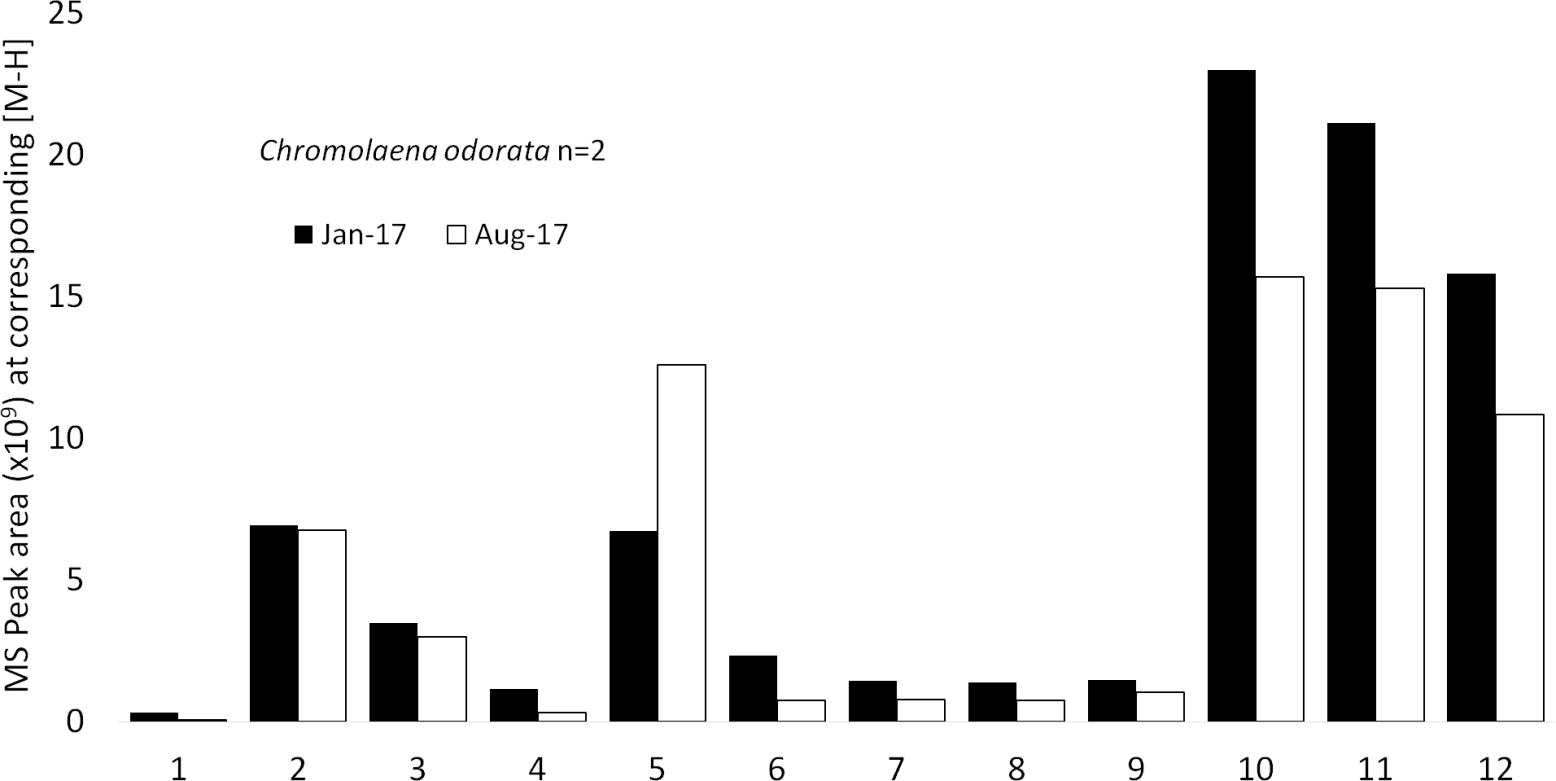
Quantitative comparison of compounds eluted from *Chromolaena odorata* RAMES libraries following 4 and 12-month storage at -20°C. Mean peak areas (n = 2). Kaempferol-3-O-rutinoside (1), Quercetin-disaccharide (2), Quercetin-trisaccharide (3), Rutin (4), Chlorogenic acid derivative (5), Kaempferol-3-O-glucoside (6), Methoxyhesperetin-1 (7), Methoxyhesperetin-2 (8), Methoxyhesperetin-3 (9), Sakuranetin (10), Chromomoric acid (11), Oxophyto-9,15-dienoic acid (12).

RAMES samples from fully-developed leaves of three invasive plant species were collected at Kruger National Park (KNP), South Africa, in mid-August 2016: (1) *Chromolaena odorata* (L) R.M.King & H.Rob, (2) *Datura stramonium* L., and (3) *Datura inoxia* Mill. These species were selected because their distribution at KNP was well mapped and their secondary metabolites were well-described and easily quantifiable. Permit for their collection was obtained from KNP (Permit number: RASI1343). Duplicate samples were collected from each plant–two different plants for each species. In mid-January 2017 (4 months after collection and storage at -20°C), one duplicate RAMES sample from each plant was analyzed with UPLC/MS (see next section for methods). In Mid-August 2017 (12 months after collection and storage in -20°C) the second set of duplicate discs was analyzed using identical conditions. For UPLC/MS analysis, RAMES discs were eluted in 50 ml of 70% ethanol on a shaker overnight. The extract was dried in a vacuum centrifuge, weighed, and resuspended in 70% ethanol to a concentration of 5 mg dried extract /ml ethanol to normalize the concentration of solids across all samples before UPLC/MS analysis. Twelve compounds were putatively identified in RAMES samples from *C*. *odorata* (Fig 3, S1 Fig). After 12 months of storage, the same compounds were still present in the eluate at the same ratio as they were after 4 months of storage, although some reduction in the levels of most compounds was observed.

**Fig 4:**
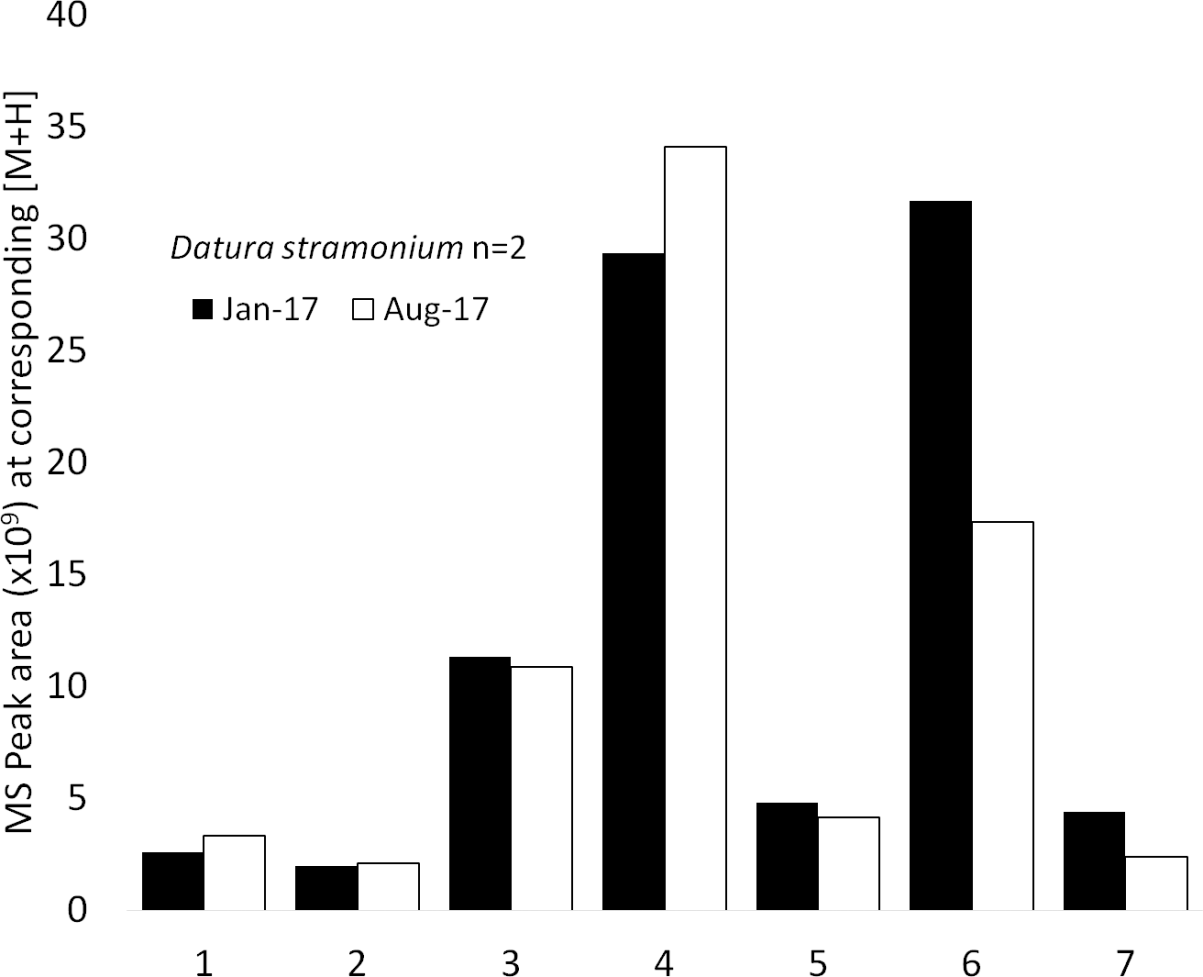
Quantitative comparison of compounds eluted from *Datura stramonium* RAMES libraries following 4 and 12-month storage at -20°C. Mean peak areas (n = 2). 6-Hydroxy-hyoscyamin (1), 6-Hydroxy-hyoscyamin-2 (2), Scopolamin (3), Atropine (4), 3-Tigloyloxy-6,7-dihydroxytropane (5), Daturilin (6), Hydroxy-oxowithatrienolide (7).

Out of seven compounds detected in the *D*. *stramonium*, two showed some degradation; daturilin showed the largest, with a 45% reduction in content (Fig 4, S2 Fig).

**Fig 5:**
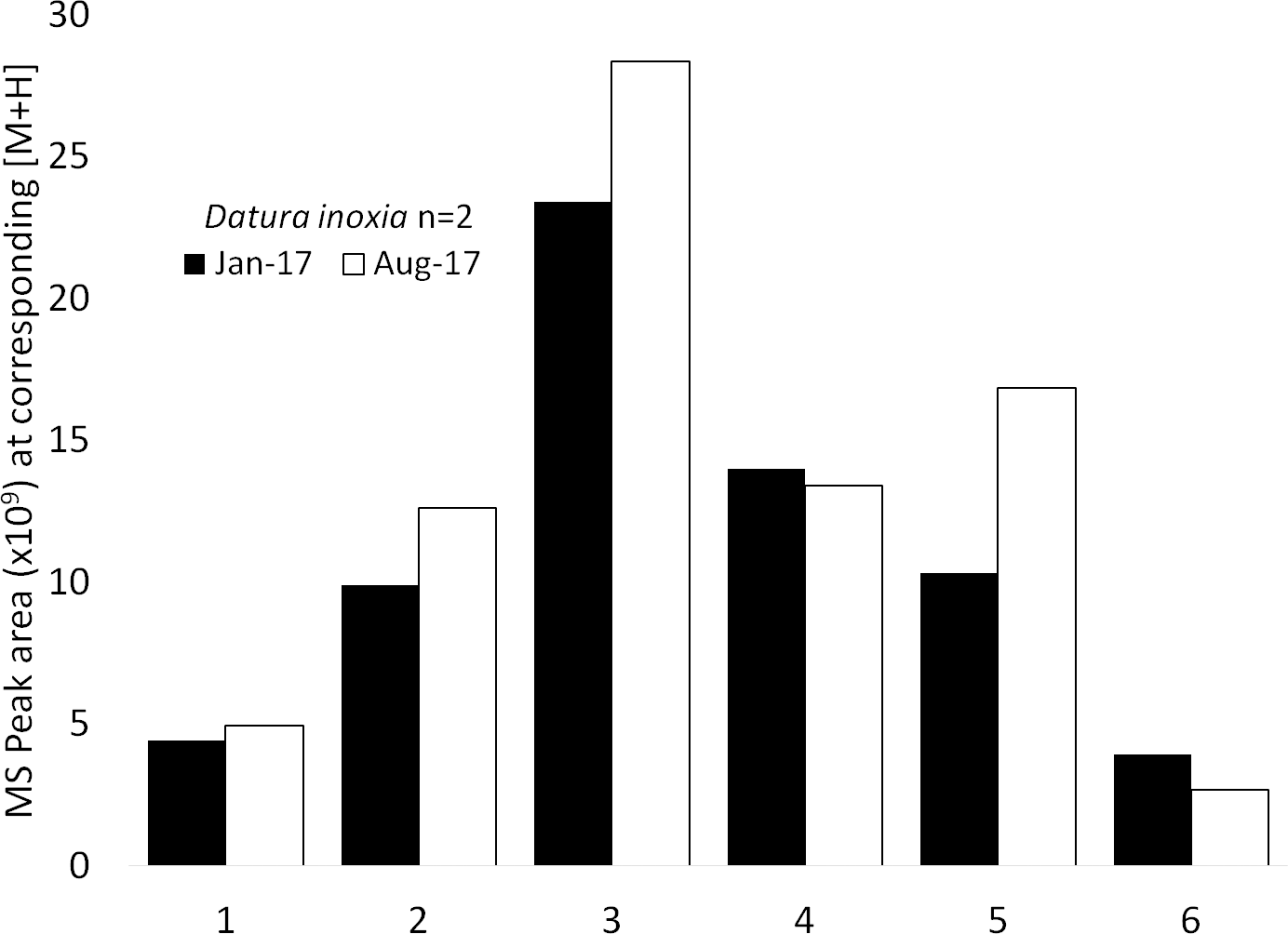
Quantitative comparison of compounds eluted from *Datura inoxia* RAMES libraries following 4 and 12-month storage at -20°C. Mean peak areas (n = 2). 6-Hydroxy-hyoscyamin (1), Scopolamin (2), Atropine (3), 3-Tigloyloxy-6,7-dihydroxytropane (4), Daturilin (5), Hydroxy-oxowithatrienolide (6).

Out of six compounds putatively identified in *D*. *inoxia* none have shown considerable degradation (Fig 5, S3 Fig). The content of four compounds increased, possibly due to experimental error or biotransformation from precursors.

Demonstration of the effective elution of compounds from GF/D discs was performed with RAMES samples from fully developed leaves of *Coffea arabica* L., *Theobroma cacao* L., *Moringa oleifera* Lam., and *Artemisia dracunculus* L. grown in Rutgers University greenhouses. These plants were selected because they contain well-defined classes of secondary metabolites that could be detected with the UPLC/MS analysis employed for this study. These species were also subsequently tested in the functional anti-microbial assays. Extracts from these plants were prepared with the standard RAMES extraction protocol (Fig 1). These extracts were either subjected to metabolome analysis directly or sorbed onto GF/D discs and eluted after 14 days of storage at -20°C, prior to UPLC/MS analysis (Table 2, S4–S7 Figs). Elution was done in 70% ethanol, as previously described. Extracts measured directly, without being sorbed to glass fiber discs, were dried in a vacuum centrifuge and resuspended in 70% ethanol to a concentration of 5 mg dried extract /ml in order to normalize the concentration of solids before UPLC/MS analysis.

**Table 2:** Quantitative comparison of compounds sorbed and then eluted from RAMES discs to compounds present in the initial extract (non-sorbed). Fully expanded leaves of *Coffea arabica*, *Theobroma cacao*, *Artemisia dracunculus*, and *Moringa oleifera* were extracted in 5 ml of ethanol as described in Fig 1 and either sorbed to glass fiber discs and then eluted with 70% ethanol or analyzed directly by the UPLC/MS (see above). Values represent the mean extracted ion chromatogram peak areas at the corresponding m/z value and ionization mode (x10^8^) for each compound putatively identified in the extract ±S.D. (n = 4). Statistical significance was calculated with an unpaired, two-tailed T-test with Welch's correction. Number of asterisks define statistical significance: * p < 0.05, ** p < 0.01, *** p < 0.001, **** p < 0.0001. NSD, No significant difference at p > 0.05. Abbreviations: PACs, proanthocyanidins; DMC, dihydroxy-methoxy chalcone; MIC, moringa isothiocyanate. M/z values for MICs, glucosinolate and niazirin represent [M-H] + acetic acid ion adducts. Quercetin and kaempferol glycosides were identified based on their [M-H] molecular ions and quantified based on [M+H] aglycone MS/MS fragments at (+) ESI MS ionization. Representative total ion current chromatograms of eluates and extracts can be viewed in S4–S7 Figs.

Plant Name	Putative compound	m/z value (ESI-MS) ionization mode		Peak area: Eluted from RAMES discs	Peak areas: Original extract, not sorbed	Significance
		(-)	(+)			
*Coffea arabica*	Caffeine		195	192.03 ± 11.79	174.03 ± 10.62	NSD
	PACs-Trimer	865		4.29 ± 0.76	6.28 ± 0.53	*
	PACs- Dimer	577		9.45 ± 0.49	10.91 ± 0.43	**
	Catechin derivative	451		18.99 ± 1.07	18.68 ± 1.48	NSD
	Catechin caffeate	451		19.82 ± 6.80	20.87 ± 1.55	NSD
	Neochlorogenic acid	353		14.26 ± 1.18	12.11 ± 1.26	NSD
	Chlorogenic acid	353		245.10 ± 10.80	222.33 ± 2.76	*
*Theobroma cacao*	Kaempferol-3-glycoside	447	287	0.70 ± 0.35	1.16 ± 0.05	NSD
	Kaempferol		287	1.40 ± 0.13	1.71 ± 0.11	*
	Kaempferol derivative		287	0.54 ± 0.05	0.46 ± 0.03	NSD
	Isoscutellarein glucuronide	461		12.73 ± 1.04	12.64 ± 0.43	NSD
	Caffeoyl L-DOPA		360	2.93 ± 0.46	59.05 ± 2.13	****
	Caffeoyl tyrosine		344	5.59 ± 0.18	24.07 ± 1.20	****
	Dihydroxy-cinnamoyl tyrosine		328	17.09 ± 1.31	21.42 ± 1.33	**
*Artemisia dracunculus*	6-Demethoxy capillarisin	285		8.47 ± 5.60	15.09 ± 6.10	NSD
	Sakuranetin	285		51.34 ± 13.03	57.07 ± 24.98	NSD
	DMC-1	271		14.62 ± 2.74	12.49 ± 5.56	NSD
	DMC-2	271		63.59 ± 10.69	53.09 ± 20.12	NSD
	Neochlorogenic acid	353		2.39 ± 0.82	5.29 ± 0.29	**
	Chlorogenic acid	353		31.31 ± 8.77	53.00 ± 3.86	*
	Isochlorogenic acid	353		3.34 ± 0.81	3.58 ± 0.13	NSD
	Dicaffeoyl quinic acid	515		4.98 ± 2.20	9.53 ± 1.62	*
	Tetrahydroxy-methoxy flavone (1)	315		8.50 ± 3.13	3.25 ± 1.23	NSD
	Tetrahydroxy-methoxy flavone (2)	315		3.50 ± 2.74	6.48 ± 2.78	NSD
	Davidigenin	257		10.47 ± 5.66	15.91 ± 6.94	NSD
*Moringa oleifera*	MIC-1	370		146.20 ± 14.71	64.37 ± 52.74	NSD
	MIC-2/3	412		11.63 ± 1.34	3.71 ± 2.72	**
	MIC-4	412		70.72 ± 7.84	35.69 ± 28.46	NSD
	Glucosinolate	612		18.30 ± 3.98	6.30 ± 2.39	**
	Niazirin	338		4.65 ± 0.82	8.01 ± 1.89	*
	Quercetin-3-glycoside	463	303	0.20 ± 0.11	135.22 ± 233.29	NSD
	Quercetin-3-malonyl-glycoside	549	303	0.13 ± 0.07	0.28 ± 0.01	*
	Kaempferol-3-glycoside	447	287	0.13 ± 0.03	0.15 ± 0.01	NSD
	Kaempferol-3-malonyl-glycoside	533	287	0.15 ± 0.05	0.21 ± 0.01	NSD
	Kaempferol-3-malonyl-glycoside isomer	533	287	0.05 ± 0.01	0.07 ± 0.01	NSD
	Neochlorogenic acid	353		0.22 ± 0.09	0.49 ± 0.17	NSD
	Isochlorogenic acid	353		1.07 ± 0.50	3.38 ± 1.28	*
	Chlorogenic acid	353		0.15 ± 0.06	0.45 ± 0.16	*

Out of seven structures putatively identified in the extract of *C*. *Arabica*, four showed no significant difference in content between sorbed (RAMES disc-eluted) and non-sorbed extracts. Two were higher in the non-sorbed extract, and one was higher in the sorbed extract. Out of seven structures putatively identified in the extract of *T*. *cacao*, three were not significantly different, while four were present in higher concentrations in the non-sorbed extract. Out of 11 structures present in the extract of *A*. *dracunculus*, eight were not significantly different in sorbed and non-sorbed extracts, while three were present in the higher level in the non-sorbed extract. Finally, out of 13 structures putatively identified in *M*. *oleifera*, seven were not different between sorbed and non-sorbed extracts, four were higher in the non-sorbed extract, while two were higher in sorbed extract. Overall, from the 38 structures putatively identified in four tested plant species, 22 (58%) showed no significant difference in content when RAMES disc-eluted and no-sorbed extracts were compared at p < 0.05.

### Anti-microbial assays

We modified Screens-to-Nature (STN) bioactivity assays developed by Global Institute for BioExploration [23–27], to be fully compatible with the RAMES format. STN technology relies on low cost, simple, field-deployable assays to detect pharmacologically active compounds from plants and other natural sources. STN assays do not require sterile conditions or wet laboratories. They were developed to enable and empower scientists, students, and interested community members to explore their local biodiversity to discover or validate natural sources of pharmacologically active compounds. We have conducted STN training in eighteen different countries, including the US. Here, we describe four STN assays (two anti-fungal, one anti-bacterial and one anti-oxidant) modified and formatted to be seamlessly compatible with RAMES technology and 10 mm GF/D glass fiber discs.

### RAMES-STN anti-fungal assays

*Fusarium* spp. and baker’s yeast (*Saccharomyces cerevisiae*) are used as model organisms for the assays. All anti-fungal RAMES-STN assays are carried out in 24-well plates that comfortably accommodate a 10 mm GF/D disc in each well. *Fusarium inoculum* is administered by placing a 2 mm plug, punched from the plate with the potato-derived *Fusarium* agar/dextrose culture, inside each well 3–5 min after the RAMES disc.

**Fig 6:**
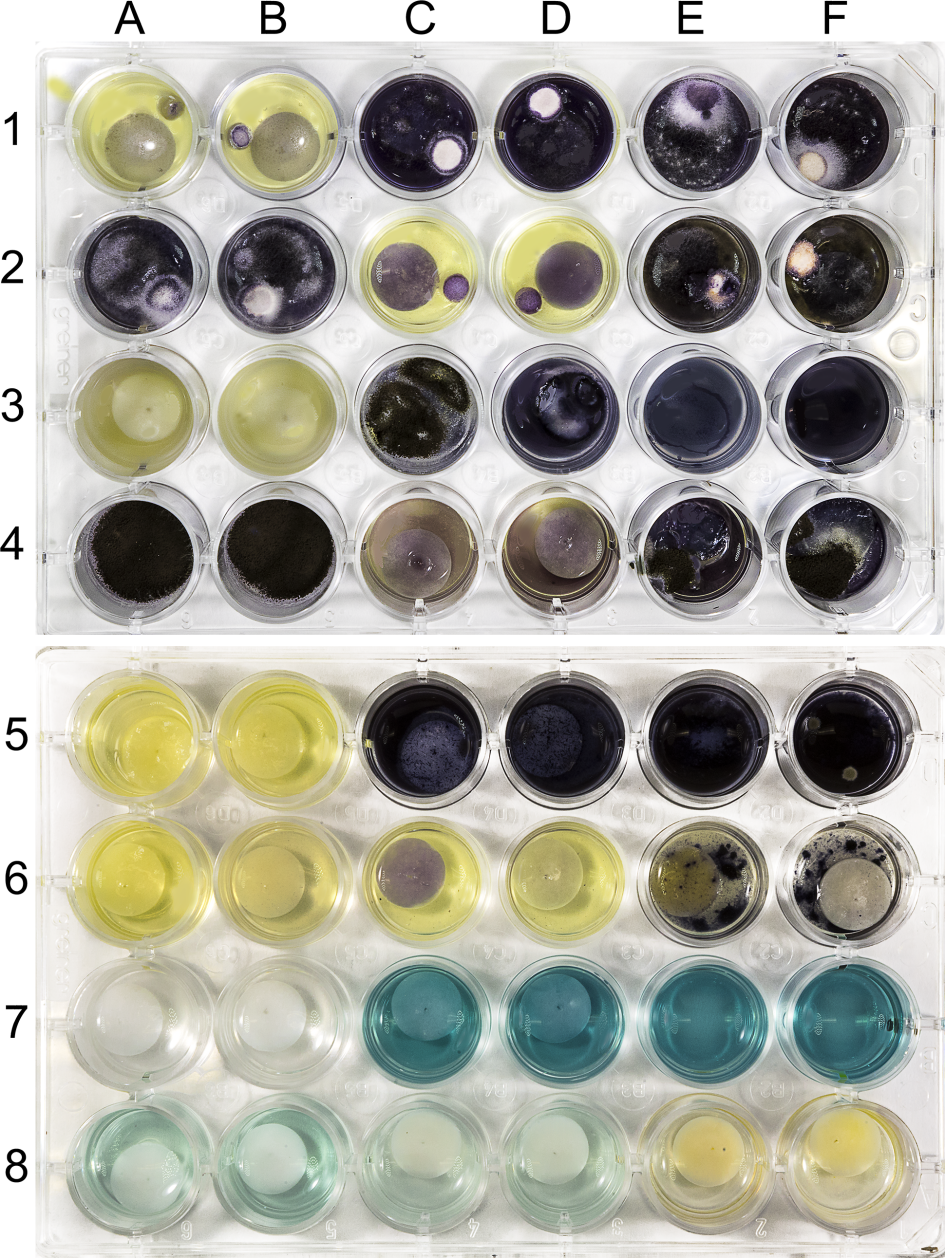
Illustration of anti-fungal, anti-bacterial and anti-oxidant RAMES-STN assays in 24-multiwell plate format. Activity is rated using a 0–3 scale with 0 representing no activity and 3 representing high activity. Activity ratings are included in parenthesis after well numbers. **Rows 1–2. *Fusarium spp*. anti-fungal assay.** Wells A1-B1 (3) positive control (glass fiber disc with econazole nitrate); C1-D1 (0) negative control (glass fiber disc with 70% ethanol); E1-F1 (0) blank (no glass fiber disc); A2-B2 (0) peeled lemon extract; C2-D2 (3) peeled garlic extract, E2-F2 (0) orange rind extract. **Rows 3–4. *Saccharomyces cerevisiae* anti-fungal assay.** Wells A3-B3 (3) positive control (econazole nitrate); C3-D3 (0) negative control; E3-F3 (0) blank; A4-B4 (0) peeled lemon extract; C4-D4 (2) peeled garlic extract; E4-F4 (0) orange rind extract. **Rows 5–6. Saliva-inoculated anti-bacterial assay.** Wells A5-B5 (3) positive control (penicillin); C5-D5 (0) negative control, E5-F5 (0) blank; A6-B6 (3) peeled lemon extract; C6-D6 (3) peeled garlic extract; E6-F6 (1) orange rind extract. **Rows 7–8. Anti-oxidant assay.** Wells A7-B7 (3) positive control (ascorbic acid); C7-D7 (0) negative control; E7-F7 (0) blank; A8-B8 (1) peeled lemon extract; C8-D8 (2) peeled garlic extract; E8-F8 (3) orange rind extract.

For the yeast assays, 600 *μ*l of the yeast inoculum solution is pipetted into each well containing the RAMES disc. A GF/D disc impregnated with 90 *μ*l of econazole nitrate solution (300 mg/ml dimethyl sulfoxide) is used as a positive control for both fungal assays; a GF/D glass fiber disc loaded with 90 *μ*l of 70% ethanol was used as a negative control. To test the anti-fungal activity of plant extracts, plates are incubated for 72 h at room temperature. To accelerate fungal growth and reduce the assay time to 48 h, plates can be incubated at 37°C to enhance fungal growth. All treatments are duplicated in adjacent wells (Fig 6).

In the field, results observed following the viability staining with MTT are recorded on a 0–3 qualitative scale, with 3 denoting the highest activity (light yellow, similar to positive standard), and 0 indicating no activity (dark purple, similar to negative control). Activity can be more precisely quantified by spectrophotometric measurements of absorbance value of the solution within each well using a test wavelength and reference wavelength at 570 nm and 630 nm, respectively [28]. For some extracts, MTT may stain RAMES discs purple, possibly indicating some chemical reaction between the stain and sorbed phytochemicals (Fig 6, wells 2C-D, 6C). This staining is not related to anti-microbial activity and can be ignored, while the true activity is determined by color changes of solution in the well.

RAMES-STN anti-fungal assays are illustrated in Fig 6 (wells A1 to F4) using RAMES discs prepared from locally purchased, peeled lemons (*Citrus limon* L.), peeled garlic cloves (*Allium sativum* L.), and orange (*Citrus sinensis* L.) rinds.

### RAMES-STN anti-bacterial assay

Growing conventional bacterium inoculum requires significant time, sterile conditions, and special equipment (i.e. autoclaves, sterile glassware and sterile transfer hoods). These requirements are difficult to fulfill in the field or in a basic laboratory. In the field conditions, human saliva makes an excellent bacterial inoculum of easily culturable bacteria for RAMES-STN screens. For anti-bacterial assays, 600 μl of saliva inoculum in LB broth is added to each well containing RAMES discs and plates incubated for 48 h at room temperature or at 37°C. MTT-based anti-bacterial activity detection is identical to that described for the STN anti-fungal assays. RAMES-STN anti-bacterial assays are illustrated in Fig 6 (wells A5-F6) using RAMES discs prepared from locally purchased, peeled lemon (*Citrus limon* L.), garlic cloves (*Allium sativum* L.) and orange (*Citrus sinensis* L.) rinds. Penicillin (90 *μ*l of 4 mg/ml solution loaded onto GF/D disc) is used as positive control.

### Detection of anti-microbial activity with RAMES-STN assays

Thirty two plants, purchased in local grocery stores or collected at Rutgers University greenhouse, were assayed for anti-microbial activities using the RAMES-STN methodology (Table 3). Traditional uses as anti-microbial medicinal plants or spices were the main criteria for choosing plant material for testing. Only two plants did not have activity in any of the three screens, scoring “0” (peace lily and spearmint), 17 plants showed at least one “3” (highest) activity, and 7 plants scored “3” in all three screens (clove, juniper berries, pomegranate, star anise, tamarind, tarragon and upland cress).

**Table 3:** RAMES-STN evaluation of 32 plants for their antimicrobial activities.

Common name	Latin name	Tissue part	Bacteria	Fusarium	Yeast
Ajowan caraway	*Trachyspermum* *ammi*	Seeds	0	3	3
Arabian coffee	*Coffea arabica*	Fresh leaves	1	0	1
Avocado	*Persea americana*	Fresh peel	1	0	1
Black cardamom	*Amomum subulatum*	Dry fruit	1	1	1
Black pepper	*Piper nigrum*	Dry unripe fruit	1	0	3
Clove	*Syzygium aromaticum*	Flower bud	3	3	3
Cocoa	*Theobroma cacao*	Fresh leaves	0	0	1
Dill	*Anethum graveolens *	Seeds	2	2	3
Grain of paradise	*Aframomum melegueta *	Seeds	1	0	0
Horseradish tree	*Moringa oleifera *	Fresh leaves	1	3	2
Indian bay leaf	*Cinnamomum tamala*	Dry leaves	2	0	1
Indian gooseberry	*Phyllanthus emblica*	Frozen fruit	3	1	3
Indonesian cinnamon	*Cinnamomum burmannii*	Dry bark	3	1	3
Juniper berries	*Juniperus communis*	Dry seed cone	3	3	3
Longan	*Dimocarpus longan *	Fresh peel	2	0	2
Malanga	*Caladium colocasia*	Fresh leaves	0	1	0
Mountain ironwort	*Sideritis montana*	Dry leaves and flowers	3	0	3
Nutmeg	*Myristica fragrans *	Seeds	2	0	1
Peace lily	*Spathiphyllum wallisii*	Fresh leaves	0	0	0
Pomegranate	*Punica granatum *	Fresh peel	3	3	3
Porcelainflower	*Hoya carnosa*	Fresh leaves	1	2	3
Quinoa	*Chenopodium quinoa*	Leachate	2	1	2
Rambutan	*Nephelium lappaceum*	Fresh peel	3	0	3
Redstem wormwood	*Artemisia scoparia*	Fresh leaves	0	1	2
Roselle	*Hibiscus sabdariffa*	Dry flower	3	3	3
Spearmint	*Mentha spicata*.	Fresh leaves	0	0	0
Star anise	*Illicium verum*	Dry flower	3	3	3
Sweet basil	*Ocimum basilicum*	Fresh leaves	0	0	1
Sweet fennel	*Foeniculum vulgare*	Seeds	1	0	3
Tamarind	*Tamarindus indica *	Pods	3	3	3
Tarragon	*Artemisia dracunculus*	Fresh leaves	3	3	2
Upland cress	*Barbarea verna*	Seeds	3	3	3

### RAMES-STN anti-oxidant assay

Anti-oxidant compounds in plants have been historically associated with health benefits [29–31]. Phenolics, carotenoids and organic acids are the main groups of anti-oxidants in plants. These compounds have been extensively studied for their pharmacological activities against chronic diseases and cancer [32]. To measure anti-oxidant activities in RAMES libraries, RAMES discs are added to each well immediately before the ABTS assay solution is dispensed into each well, and results recorded after 2 h of incubation at room temperature with some agitation. Anti-oxidant activity is visually determined on a 0–3 scale according to solution color change. Samples lacking anti-oxidant activity remain blue and score 0, while samples with high anti-oxidant activity turn colorless and score 3. Scoring is done according to the color of the solution, not the glass fiber disc. If more precise quantification is required, spectrophotometer can be used to record the absorbance value at 734 nm.

RAMES-STN anti-oxidant assays are illustrated in Fig 6 (wells A7 to F8) using RAMES discs prepared from locally purchased, peeled lemons (*Citrus limon* L.), peeled garlic cloves (*Allium sativum* L.) and orange (*Citrus sinensis* L.) rinds.

## Discussion

Plant metabolome research offers significant benefits to human health and wellness, the global environment, and fundamental science [33–35]. However, collecting samples of plant metabolome from plants, particularly those grown in the wild, can be laborious and complicated, involving destructive harvesting and processing of large amounts of plant materials through lengthy extraction and drying processes. Long processing times with large volumes of solvents often leads to degradation of bioactive compounds in addition to creating problems with transportation and storage.

Miniaturized RAMES technology, described and validated in this manuscript, offers a cost- and time-effective alternative to conventional collection of plant extracts. RAMES allows compact storage of thousands of plant metabolome samples in a format compatible with screening strategies that utilize glass fiber discs impregnated with phytochemicals in the variety of functional assays. RAMES technology also provides a format for storing plant metabolome libraries for future biochemical studies, as most phytochemicals can be easily eluted from the discs (Table 2). We suggest that the short sample extraction and drying times offered by RAMES technology stabilizes many labile phytochemicals compared to other library collection strategies. However, direct comparative studies are needed to confirm this assumption. RAMES libraries can be collected with almost no environmental impact. A total of 2 g wet tissue (leaves, flowers) or 200 mg dry tissue (seeds, bark, wood), sufficient for preparing 25–35 replicate RAMES samples, can be harvested without significantly disrupting collection sites or source plants. Using ethanol as the only solvent may limit the diversity of the natural products extracted from plant tissues. Therefore, future users of RAMES technology may consider using solvents with different extraction properties, such as water, hexane, or acetone, to create more diverse metabolomic libraries.

At present, we can only evaluate the stability of RAMES libraries after 12 months of storage (Figs 3–5). More detailed studies over longer periods, as well as the direct comparison of RAMES technology to conventional methods of storing dried extracts, are needed to fully address stability concerns associated with storage of any biochemical samples. However, the initial data from samples eluted from RAMES libraries over time suggest that many compounds remain stable after 12 months of storage, while others slowly degrade or become more difficult to elute. This is not at all surprising as many phytochemicals are inherently unstable and have short half-lives, particularly in extracts [8,36]. We have also confirmed that the major phytochemicals detected in freshly prepared extracts from four plant species can be eluted from RAMES discs stored for 2 weeks at -20°C (Table 2). Measured differences in the content of some phytochemicals may be explained by their inherent instability, biotransformation during storage, or binding to glass fiber discs, which hinders elution. Overall, our data indicate that sorbing plant extracts onto glass fiber discs followed by storage in a standard freezer causes relatively minor quantitative and qualitative compositional changes compared to a fresh extract. It is reasonable to assume that storage at -80°C will further increase the storage life of RAMES libraries.

A key advantage of RAMES libraries is their immediate compatibility with functional assays that were co-developed with them, as demonstrated on the examples of anti-fungal, anti-bacterial, and anti-oxidant assays (Fig 6). These simple, field-deployable STN assays require minimal laboratory equipment and are formatted to accommodate RAMES discs removed from storage and placed inside wells of a 24-multiwell plate containing appropriate reagents and inoculum. Thus, these assays can be performed at a field collection site anywhere in the world, not requiring transport of the samples to a special screening facility that may be outside the country of origin. Such removal of samples from the country of origin may violate the 1992 Rio Convention on Biological Diversity and has historically increased the challenges of bioprospecting. For the STN anti-fungal assays that can be operational in non-sterile field conditions, we adapted *Fusarium* spp. that can be easily cultured from the surface of tuberous vegetables, and common baker’s yeast, available in most locations. Human saliva was found to be a good source of bacteria inoculum, available outside the laboratory and carried by every individual. We found a 24-multiwell plate format with liquid media in each well to be the most applicable to the basic laboratory and field conditions. However, we found agar diffusion assays [37] to be also well compatible with RAMES technology (data not shown). This assay uses the radius of the microbial inhibition zone around the compound(s)-impregnated filter disc placed on the surface of inoculated agar as a measure of antimicrobial activity. We have validated the use and predictability of RAMES-STN anti-microbial assays by testing 32 medicinal plants and spices with reported anti-microbial activities (Table 3). Thirty of these plants showed detectable activity, in at least one screen; seven showed highest level of activity (complete kill = “3” similar to positive control) in all screens i.e., one anti-bacterial and two anti-fungal. These data confirm the utility of the RAMES-STN technology for detecting anti-microbial activities.

The simplicity of STN assays not only allows rapid lead detection in the field, but also makes them excellent educational tools for biology teachers at schools and universities [23–27]. RAMES technology combined with STN assays connects students with traditional botanical knowledge and stimulates their interest in science, human health, and biodiversity conservation. We also see value in collecting RAMES libraries from regions threatened with biodiversity loss. Such cataloging of phytochemical diversity is needed to preserve the information about the ecosystems that may be lost forever.

Finally, while this manuscript only describes the application of RAMES-STN platform to plant metabolome, this technology can be just as easily adapted to collecting metabolome libraries from other lifeforms on our planet. Sharing RAMES libraries with other researchers by sending them metabolome-impregnated glass fiber discs from a species of interest should further strengthen studies of natural products and enable the development of novel functional screens compatible with this technology.

## Supporting information

S1 Fig Comparative total ion current chromatograms (MS scanning mode from m/z 100 to m/z 1000) of eluates from *Chromolaena odorata* RAMES libraries after LCMS analyses following 4 and 12 month storage at -20°C. (PDF)

S2 Fig Comparative total ion current chromatograms (MS scanning mode from m/z 100 to m/z 1000) of eluates from *Datura stramonium* RAMES libraries after LCMS analyses following 4 and 12 month storage at -20°C. (PDF)

S3 Fig Comparative total ion current chromatograms (MS scanning mode from m/z 100 to m/z 1000) of eluates from *Datura inoxia* RAMES libraries after LCMS analyses following 4 and 12 month storage at -20°C. (PDF)

S4 Fig Comparative total ion current chromatograms (MS scanning mode from m/z 100 to m/z 1000) of eluates and extracts from *Coffea arabica*. (PDF)

S5 Fig Comparative total ion current chromatograms (MS scanning mode from m/z 100 to m/z 1000) of eluates and extracts from *Theobroma cacao*. (PDF)

S6 Fig Comparative total ion current chromatograms (MS scanning mode from m/z 100 to m/z 1000) of eluates and extracts from *Artemisia dracunculus*. (PDF)

S7 Fig Comparative total ion current chromatograms (MS scanning mode from m/z 100 to m/z 1000) of eluates and extracts from *Moringa oleifera*. (PDF)
