# Supplementary material for: Rapid, field-deployable method for collecting and preserving plant metabolome for biochemical and functional characterization
Source: PLoS One. 2018 Sep 6;13(9):e0203569. doi: 10.1371/journal.pone.0203569 (PMC6126852; doi:10.1371/journal.pone.0203569)
Supplement: S7 Fig — (PDF) [file pone.0203569.s007.pdf]

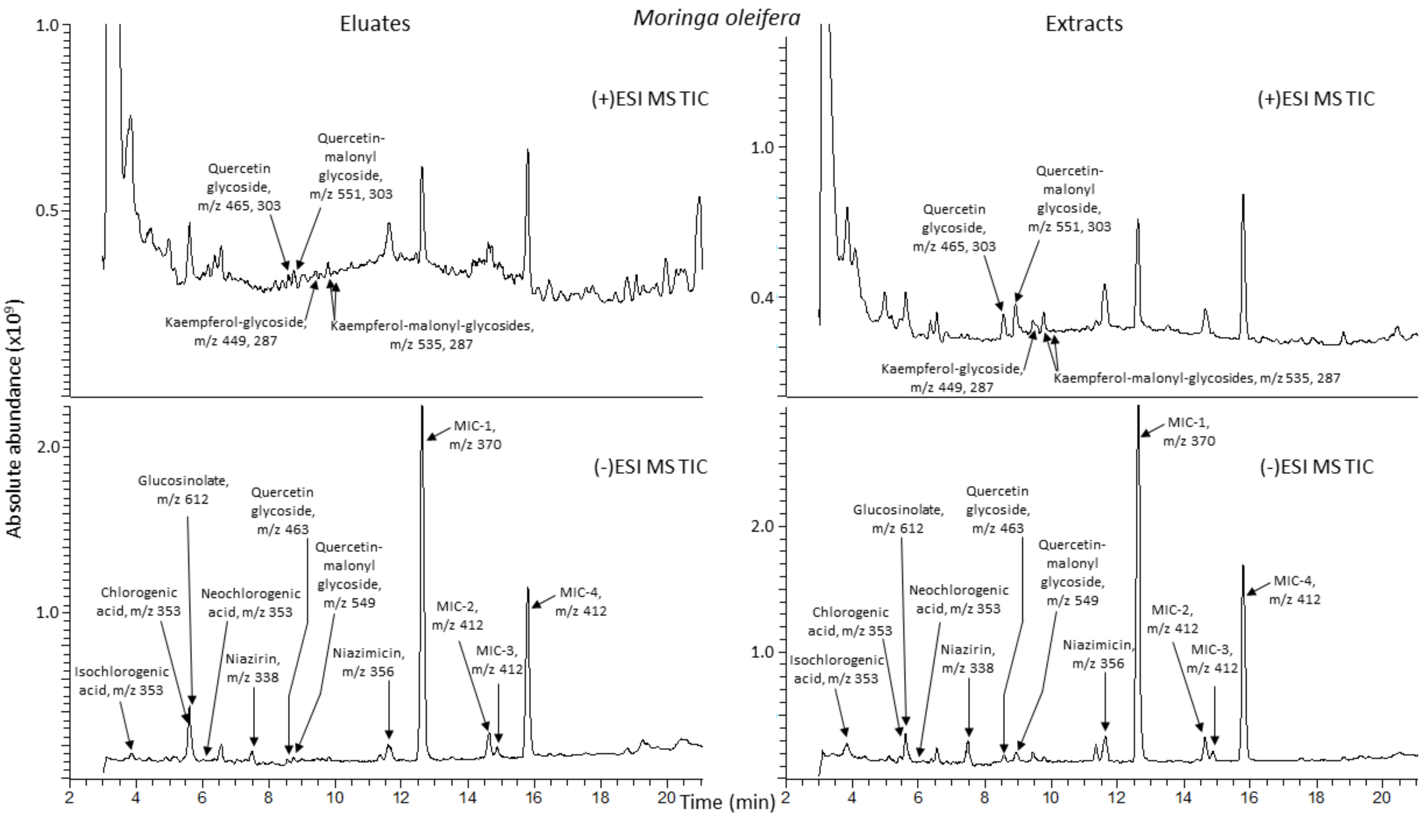

S7 Fig. Representative total ion current chromatograms (MS scanning mode from m/z 100 to m/z 1000) of eluates and extracts from *Moringa oleifera*.

Peak identification based on isotope abundance analysis of high-resolution mass spectral data and reporting the best fitting empirical formula, as well as retention time of available commercial standards.
